# Using Information and Communication Technologies (ICT) for Mental Health Prevention and Treatment

**DOI:** 10.3390/ijerph18020461

**Published:** 2021-01-08

**Authors:** Ana Fonseca, Jorge Osma

**Affiliations:** 1 Center for Research in Neuropsychology and Cognitive-Behavioral Intervention (CINEICC), Faculty of Psychology and Educational Sciences, University of Coimbra, 3000-115 Coimbra, Portugal; 2 Health Research Institute of Aragon, C/San Juan Bosco, 13, 50009 Zaragoza, Spain; 3 Department of Psychology and Sociology, Universidad de Zaragoza, C/Atarazanas, 4, 44003 Teruel, Spain

Mental disorders are a recognized population health issue, with recent estimates placing mental illness as the first in global burden of disease in terms of years lived with disability, and comparable to cardiovascular and circulatory diseases in terms of disability-adjusted life years. Common mental disorders refer to a range of anxiety and depressive disorders, which are prevalent disorders around the world (4.4% and 3.6% of the global population suffer from depression and anxiety disorders, respectively), with variations across different regions and populations [1]. Despite the human, social, and economic costs of mental disorders, mental health has been often neglected. Recently, the Lancet Commission on Global Mental Health and Sustainable Development launched a report recommending that mental health should be reframed as a fundamental human right, and that the definition of mental health should be expanded to promote mental wellbeing, prevent mental health problems, and enable recovery from mental disorders.

Despite this, we still face great inequalities in access to mental health promotion, prevention, and treatment programs worldwide (e.g., limited resources to accommodate the existent needs) and/or treatment uptake barriers, such as attitudinal barriers (e.g., stigma towards mental health) or structural barriers (e.g., geographical or financial restrictions, work constraints, or patient’s physical conditions). The delivery of psychological services—including assessment/monitoring, mental health promotion, prevention, and treatment—through information and communication technologies (ICT) may be an effective way of improving individual access and use of mental healthcare services [2]. ICT-delivered psychological services include (but are not restricted to) web-based interventions, mobile apps, videoconferencing systems (telepsychology), or virtual reality systems, and may be used complementary to face-to-face services or as the sole means of access to psychological interventions. The use of ICT-delivered psychological services has several advantages, related with increased accessibility and flexibility, self-monitoring integrated into treatment, and empowerment promotion, as well as increased novelty and appeal. However, we cannot exclude that they also have challenges and limitations, related with low digital literacy or with safety and privacy issues, among others [2].

The present Special Issue focuses on acceptability, cost-effectiveness, potentialities, and limitations of ICT-based psychological services for mental health promotion, prevention, and treatment. Sixteen articles are included in this special issue from different international research teams working in China, Portugal, Spain, and the United States.

Five of the studies included in this Special Issue focused on the assessment of ICT tools for the prevention [3,4] and treatment [5,6] of mental disorders, or the promotion of mental health [7], in different populations. Specifically, these studies examined the interventions’ effectiveness, clinical utility, acceptability, or feasibility through pilot trials or randomized controlled trials.

Two of the studies focused on the adolescent population. Gladstone et al. [3] examined the effects of an online preventive intervention for depression disseminated through primary care settings targeting adolescents at moderate-to-high risk for depression (Competent Adulthood Transition with Cognitive-behavioral, Humanistic, and Interpersonal—CATCH-IT), compared with a health education online program. Although long-term reductions of depressive symptoms were found in both groups, participants in the CATCH-IT group also showed a cross-over effect for anxiety symptoms, suggesting the potential for transdiagnostic interventions targeting underlying mechanisms shared by both disorders. Moreover, the study showed that the presence of some pre-conditions (e.g., supportive environmental factors such as a supportive relationship with a physician and adequate paternal monitoring) may favor the benefits of the CATCH-IT intervention among adolescents. On the other hand, Sandín et al. [5] performed a pilot trial where they examined the feasibility, acceptability, and clinical utility of the internet-delivered version of the Unified Protocol for Transdiagnostic Treatment of Emotional disorders in Adolescents (UP-A). The internet-delivered version of the UP-A showed high feasibility and acceptability, with all participants and responsible parents reporting an improvement in the adolescents’ ability to cope with emotions. Moreover, the results of the pilot trial were also promising, showing preliminary evidence of the program’s efficacy in improving outcomes (anxiety and depression) and vulnerability of transdiagnostic mechanisms.

An additional study focused on the prevention of depression among non-professional caregivers at risk for depression, which are an often-neglected group, despite the high burden and negative consequences of the caregiving responsibilities. Otero et al. [4] conducted a pilot study to examine the efficacy and feasibility of a brief cognitive-behavioral intervention for depression prevention delivered through a smartphone app combined with positive and corrective feedback provided by a psychologist through conference calls. The results provided preliminary evidence of the efficacy of the intervention in reducing depressive symptoms and the risk of developing depression, both at postintervention and in the follow-up assessments, and show good indicators of adherence and satisfaction with the intervention, encouraging further research on the topic.

Finally, two other studies targeting different populations investigated the effectiveness of interventions by considering, as primary outcomes, not mental illness indicators (e.g., depression and anxiety symptoms), but positive mental health indicators (positive affect and positive mental health). Vara et al. [6] provided us with a secondary analysis of a randomized controlled trial that assessed the efficacy of a low-intensity internet intervention aimed to promote positive affect in depressive patients in primary care, as an adjunct therapy to improved treatment as usual. The results of this study supported not only that the promotion of positive affect might have an impact on the decline in clinical symptomatology and improve positive functioning, but that both the improvements in depression and in positive affect can be responsible for the long-term changes in wellbeing. Moreover, the profile of patients who can benefit most from the intervention was analyzed. Monteiro et al. [7] conducted one of the first trials examining the effectiveness, acceptability, and feasibility of a self-guided web-based intervention that was originally developed for preventing postpartum depression among high-risk women, in promoting positive mental health among women presenting low risk for postpartum depression, as even those women face significant challenges to their adjustment and may benefit from mental health promotion interventions. The results showed that women in the intervention group presented a larger increase in positive mental health compared to women in the waiting-list control group, with a large proportion of women showing good acceptability of the intervention.

Moreover, focusing on a different context (organizational context), Da et al. [8] explored, through a randomized controlled trial design, the effectiveness and feasibility of a brief online intervention (20 min during 5 days) to enhance psychological capital in workplaces (PsyCap intervention). Participants randomized to the experimental condition received daily online links with some lectures to read and activities to practice regarding how to develop efficacy, hope, resilience, and optimism. Those in the placebo condition were asked to write down self-reflections during the same timeline. Nothing was sent to the control group. The preliminary results have shown increases in psychological capital, job satisfaction and reductions in turnover intention after the intervention and at one-week follow-up.

In addition to effectiveness studies, this Special Issue includes the description of two study protocols using internet. The first one, by Branquinho et al. [9], is a two-arm, non-inferiority randomized controlled trial comparing a cognitive behavioral blended intervention to usual treatment for postpartum depression provided in healthcare centers. The blended condition will integrate face-to-face sessions with a web-based program called Be a Mom. This approach has the advantage of being dynamic and flexible, given that it allows using technology for motivating, monitoring, giving support, and treating patients, but without losing treatment sessions face-to-face. The study will explore the efficacy and cost-effectiveness of both conditions, and will offer greater support to evidence-based psychological treatments for postpartum depression. The second one, presented by Quilez et al. [10], is a pilot study with a repeated single-case experimental design (multiple baseline design) in a public mental health unit in Spain. The main focus is to further explore the clinical utility and feasibility of an online transdiagnostic emotion regulation-based intervention (the Unified Protocol) delivered in group format to people waiting for bariatric surgery with emotional symptoms or disorders. Expected outcomes will be reductions in anxiety and depression symptoms and weight maintenance over the two years follow-up period after the intervention.

Two of the studies included in this Special Issue focus on how ICT tools can be helpful in the screening and monitoring of different symptoms. Suso-Ribera et al. [11] examined if the Monitor de Dolor app—which allows daily assessment of pain through ecological momentary assessments—would be an effective option for patient monitoring, in terms of improvement of chronic musculoskeletal pain management. The authors performed a randomized controlled trial in which they compared three conditions: the usual monitoring method (onsite retrospectively), usual monitoring plus app without alarms, and usual monitoring plus app with predefined alarms sent to clinicians to make treatment adjustments. In addition to allowing the collection of patients’ pain-related data in an automated way, the results of this study were suggestive of the effectiveness of telemonitoring in pain and other mental health outcomes (e.g., depressive symptoms), particularly if an alarm system that allow changes in pain management can be implemented. Andreu-Pejó et al. [12] examined a sample of 85 pregnant women who participated in a web-based platform HappyMom (Mamáfeliz), which longitudinally assessed a set of risk factors for the development of perinatal emotional disorders. The results showed that certain personality characteristics (e.g., high neuroticism) may be a risk factor to pregnant women’s wellbeing deterioration and therefore should be assessed early during pregnancy mental health screening, which might be facilitated through the use of ICT tools. In a related topic, Gual-Montolio et al. [13] presented a systematic review about the use of ICTs for routine outcome monitoring (ROM) and measurement-based care (MBC) in face-to-face psychological interventions for mental health problems. The eighteen articles revised showed that handheld technologies such as smartphone apps, tablets, or laptops were used for assessment and feedback during psychological interventions (including ROM and MBC), providing evidence about their feasibility and acceptability.

The remaining five studies cover different aspects related to ICTs. Considering the association between therapeutic alliance to treatment outcomes in psychotherapy and the increased use of internet-based interventions, Herrero et al. [14] adapted The Working Alliance Inventory for online interventions (WAI-TECH-SF), based on the WAI Short Form [15]. Authors found good psychometric properties of the WAI-TECH-SF and its association with positive therapeutic outcomes (changes in depressive symptoms) and satisfaction with the treatment in a sample of 193 participants with depressed diagnosis.

Castilla et al. [16] studied the needs of 28 participants (58–95 years old) with a diagnosis of mild cognitive impairment, regarding an ICT-based intervention tool design for elderly care. Interesting results were found about the need to place main interaction elements in the center of the screen instead of in the peripheral areas, and also that speed of audio help had a significant impact on performance. Other usability recommendations for this specific sample are described in the article.

ICTs have changed different aspects of our daily life; one of them is the way we interact with other people searching for a romantic and/or sexual partner. In this sense, Castro et al. [17] explored, in a sample of 1705 university students, the association between present and past use of dating apps, sociodemographic data, and bright and dark personality traits. Results indicated that being men, older youth, and members of sexual minorities were more likely to be current and previous dating app users. It was not expected that dark personality showed no predictive ability. These studies are needed in order to personalize both prevention and promotion interventions of healthy romantic and sexual relationships in different target groups.

In the context of enhancing the access to a mobile-based intervention for improving maternal mood and increasing parent practices in a sample of postpartum women, Bagget et al. [18] explored the differential efficacy of three referral approaches (i.e., community agency staff referral, research staff referral, and maternal self-referral). Among the results obtained, we highlight that women who self-referred and those who were referred by community gatekeepers were as likely to eventually consent to study participation and initiate the intervention in comparison with those referred by research staff.

Finally, during the COVID-19 outbreak, mental healthcare delivery was imposed with sudden challenges. Dores et al. [19] explored changes in the delivery of psychological services through ICT during the COVID-19 pandemic. The results showed that psychologists have adopted ICTs to continue to provide mental healthcare during the COVID-19 outbreak. Despite the challenges identified, they globally assessed the experience of delivering psychological services through ICT tools as positive and with similar results, suggesting a change in attitudes towards the use of such tools.

This Special Issue bring us the ability to acknowledge some of the possibilities that ICTs in mental health offer to researchers and mental health professionals. From a clinical point of view (prevention, promotion, and treatment), as we previously explained in brief, some of the manuscripts have described different ways to solve one of the main gaps in mental health fields nowadays, that is to reach as many people in need as possible. Through ICTs, people can benefit from evidence-based prevention and treatment psychological interventions without losing their privacy, and integrating the intervention in their normal daily routine (e.g., perinatal women). As we can see, ICTs can also provide important benefits over the psychotherapy process, maintaining the therapeutic alliance and allowing clinicians to assess and get feedback of certain health or clinical variables over the interventions. Additionally, from a technology point of view (usability, feasibility, acceptability, etc.), we have seen also some examples highlighting the importance of studying the psychometric properties of measures administered through the internet, the improvements on the usability aspects of devices, especially if we want to work with special populations (e.g., elderly people), and the necessary study of users’ profiles to personalize ICTs and future interventions. Despite the limitations of the studies included in this Special Issue, in general, we can say that ICT-based interventions applied for mental health prevention or treatment have proved to be effective, feasible, and well-accepted by users. We hope that the studies, outcomes, and limitations described in this Special Issue would encourage clinicians and researchers around the world to continue working to increase the scientific evidence about the cost-effectiveness and implementation of the ICTs in mental health prevention, promotion, and treatment interventions.
